# Disentangling who is who during rhizosphere acidification in root interactions: combining fluorescence with optode techniques

**DOI:** 10.3389/fpls.2013.00392

**Published:** 2013-10-10

**Authors:** Marc Faget, Stephan Blossfeld, Philipp von Gillhaussen, Ulrich Schurr, Vicky M. Temperton

**Affiliations:** Institute of Bio- and Geosciences, IBG-2: Plant Sciences, Forschungszentrum Jülich GmbHJülich, Germany

**Keywords:** plant roots, interaction, green fluorescent protein, pH planar optodes, rhizotrons, rhizosphere, maize, bean

## Abstract

Plant–soil interactions can strongly influence root growth in plants. There is now increasing evidence that root–root interactions can also influence root growth, affecting architecture and root traits such as lateral root formation. Both when species grow alone or in interaction with others, root systems are in turn affected by as well as affect rhizosphere pH. Changes in soil pH have knock-on effects on nutrient availability. A limitation until recently has been the inability to assign species identity to different roots in soil. Combining the planar optode technique with fluorescent plants enables us to distinguish between plant species grown in natural soil and in parallel study pH dynamics in a non-invasive way at the same region of interest (ROI). We measured pH in the rhizosphere of maize and bean in rhizotrons in a climate chamber, with ROIs on roots in proximity to the roots of the other species as well as not-close to the other species. We found clear dynamic changes of pH over time and differences between the two species in rhizosphere acidification. Interestingly, when roots of the two species were interacting, the degree of acidification or alkalization compared to bulk soil was less strong then when roots were not growing in the vicinity of the other species. This cutting-edge approach can help provide a better understanding of plant–plant and plant–soil interactions.

## INTRODUCTION

The main root functions are to ensure both uptake of water and nutrient resources as well as provide an anchorage function for the whole plant. Moreover, Darwin (1880) considered roots to act as the plant brain integrating information from multiple sources. Despite these key functions of roots for whole plant performance, root ecophysiology and ecology have until relatively recently been a field of research weighed down by seemingly unsolvable difficulties in following root growth *in situ* in natural substrates. The soil–root–rhizosphere system has until recently been considered a black box that is hard to reach and to study (Faget et al., 2013).

Roots are continuously interacting with their environment, not only with their direct abiotic environment (as in the rhizosphere), but also interacting with biotic neighbors such as roots of neighboring plants, microbes, and soil fauna (Bonkowski et al., 2009). Alone when considering root interactions with the abiotic environment in the soil, processes occur at very variable spatial and temporal scales. Recent years have shown important breakthroughs in understanding the complex interplay of how roots both react to and affect their environment (de Kroon and Mommer, 2006; Schreiber et al., 2011; de Kroon et al., 2012; Postma and Lynch, 2012). It is well documented that plant roots are able to actively alter the biogeochemistry of their vicinity, the rhizosphere (Hiltner, 1904; Hinsinger et al., 2003, 2005, 2009). This interaction of plant roots with the soil causes a highly complex spatial and temporal pattern of micro niches that are potentially characterized by large differences in, e.g., soil water content, soil pH, nutrient availability, microbial community structure and activity. There are several drivers for this interaction, but root foraging for the resources water and nutrients are of most importance. Foraging and uptake of nutrients can cause strong variations in soil pH. For example, during the uptake of nitrate or ammonium, plant roots release OH^-^ (hydroxyl ions) or H^+^ (protons) in order to maintain electro-neutrality across the root membrane (Marschner and Romheld, 1983; Colmer and Bloom, 1998; Hinsinger et al., 2003). On the other hand, plant roots are able to release large amounts of organic acids such as citric acid, in order to mobilize nutrients (e.g., phosphorous) when they are bound to soil particles and therefore inaccessible for direct uptake (Jones et al., 2003; Lambers et al., 2006). Both processes can create pH gradients of more than one pH unit from the root surface to the bulk soil. Additionally, when considering the dynamic growth of plant roots, it quickly becomes clear that the further elucidation of plant soil interactions is not a trivial task but very important for understanding plant performance, especially under stressful conditions. This point becomes even more pertinent, when the target plants are crops such as maize or bean and when the aim of the research is to sustain or even to improve the yield of crops in low-input agro-ecosystems.

When different species are sharing the same soil volume, they have to forage for the same essential resources that are often limiting and to explore and adapt to their environment to be able to uptake sufficient resources for maintaining their growth. Major advances in root research both in ecology and ecophysiology have shown that roots respond both to nutrient availability (Hodge, 2004; Cahill et al., 2010) but also to the presence of different microfauna groups in the soil (Bonkowski et al., 2009) as well as the presence of a neighboring plant (Callaway et al., 2002; de Kroon and Mommer, 2006; de Kroon, 2007). Some studies suggest that both kin recognition (Dudley and File, 2007; Dudley et al., 2013) and recognition of the genetic identity of neighbors can influence the proliferation of roots and root allocation (Gagliano et al., 2012; Fang et al., 2013). Gagliano et al. (2012) found that the identity of the neighbor affected the allocation to roots and shoots, as well as affecting germination of seeds. Such studies finding communication between plants beyond direct resource-based competition have received a number of critical responses (Klemens, 2008), but the number of studies finding evidence for such communication is on the rise (de Kroon, 2007; Gagliano et al., 2012). This is clearly a research field with ample need for further studies to back-up and test theories and outcomes, and novel methods being established will no doubt provide important new insights to the issue of the question of plant interactions and whether non-resource-based competition is important compared to resource-based competition. Our posit, is that novel combinations of non-invasive methods for studying roots (Rewald et al., 2012; Faget et al., 2013) can now provide important tools to explore rhizosphere interactions with more ease and will allow important new insights. For further validation and elucidation of these topics an approach is missing which enables us to investigate and understand *in situ* rhizosphere processes of plants in more detail, either growing alone or intercropped with plants of different species.

Although studying the dynamics of root growth is still a challenge, new methods are allowing us to follow roots *in situ* (Faget et al., 2013) and even to separate the roots of different species (Faget et al., 2009; Rewald et al., 2012). One of these methods uses fluorescent roots of genetically transformed plant species using fluorescent protein (FP; green fluorescent protein, GFP; Faget et al., 2009, 2010, 2012, or red fluorescent protein, RFP; Faget et al., 2013). At the same time, other methods have been developed to study rhizosphere-scale processes, such as pH, CO_2_, and O_2_ concentrations with the technique of planar optodes (Blossfeld and Gansert, 2012; Blossfeld, 2013). The FP method relies on the ability of genetically transformed roots to express fluorescing proteins and thus be visible at certain excitation and emission wavelengths, whereas the optode method uses indicator dyes on the planar optodes that get excited by specific light and emit characteristic fluorescence patterns in proportion to the concentration of the measured substance, e.g., H^+^. Planar optodes provide new opportunities to study rhizosphere processes *in situ* and dynamically over time (Blossfeld et al., 2011, 2013). There are several approaches for fluorescence detection and we refer the reader to the scientific literature for detailed comparison and evaluation of the advantages and disadvantages of the different approaches (Holst and Grunwald, 2001; Stahl et al., 2006; Gansert and Blossfeld, 2008). In studies where roots of different individuals (either of the same species or of another species) are interacting, however, it is often desirable to be able to identify which root within the region of interest (ROI) of the optode belongs to which species or genotype. For this reason, we hereby combined the GFP and planar optode methods in order to achieve the combined goal of following rhizosphere processes and being able to identify which species is which underground.

Within this context we asked:

(1) Whether we can combine the planar optode and the FP methods to visualize rhizosphere pH changes during root–root interactions between species, using the FP method to assign species identity to roots and the optode method to measure the rhizosphere pH changes.
(2) As a consequence we asked, whether we can localize specific rhizosphere processes and link them to specific plant species and their interactions?

We approached these questions by setting up an experiment with two plant species, maize and bean (*Zea mays* and *Phaseolus vulgaris*) with roots growing in rhizotrons either with or without close contact with roots of the other species. We measured selected ROIs within the rhizosphere of the rhizotrons using the planar optode method, and GFP maize to be able to identify which species is contributing to what extent to the specific pH measured in the intercropped rhizosphere.

## MATERIALS AND METHODS

### PLANT MATERIAL

The maize line ETH-M72_GFP_ expressing the GFP was grown alone or together with common bean (*P. vulgaris* “Fadenlose”). The maize genotype ETH-M72 was genetically transformed to include the gene for GFP (ETH-M72_GFP_). The transformation construct contains the gfp gene flanked by the ubiquitin promoter (ubi::gfp) and the nopaline synthase (NOS) terminator. It was cloned into the pUC19 vector, which contains the gene for ampicillin resistance (ampR) at the restriction sites *Spe*I and *Xba*I. The gfp gene was cloned into the cassette at the NcoI and SalI sites. The expressed GFP is reported to have a fluorescence peak between 500 and 520 nm when excited by light at 450–470 nm (Faget et al., 2009, 2010, 2012).

### EXPERIMENTAL CONDITIONS

Seeds of the two species were germinated on blotting paper before seedlings of comparable size were transplanted into rhizotrons. The rhizotrons had one side covered in plexiglass that is removable so that planar optodes can be installed on ROIs and roots growing on the surface are visible to the naked eye. The rhizotrons with dimensions of (400 mm × 200 mm × 20 mm) were filled with 2/3 soil (sieved with 4 mm mesh) and 1/3 sand (washed two times with deionized water). The soil and sand were mixed and each rhizotron received 1.3 l of mixed substrate. All rhizotrons were kept in a climate chamber (12 h light, 240 μmol m^-2^ s^-1^ PAR, 65% humidity, 24.5°C day, and 18.5°C night). All rhizotrons were placed at an angle of 30° from the vertical with black cover on the transparent side to prevent the roots from incident light and each rhizotron received 100 ml of 1/3 the full Hoagland’s nutrient solution at the start of the experiment. The rhizotrons additionally received 30 ml of 1/3 the full Hoagland nutrient solution per day. The full Hoagland nutrient solution used contained the following minerals: 5 mM KNO_3_, 5 mM Ca(NO_3_)_2_, 2 mM MgSO_4_, 1 mM KH_2_PO_4_, 0.09 mM Fe EDTA, 0.01 mM MnCl_2_, 0.001 mM CuSO_4_, 0.001 mM ZnSO_4_, 0.05 mM H_3_BO_3_, and 0.0005 mM Na_2_MoO_4_.

The aim of this study was to for the first time combine pH measurements using planar optodes with GFP methods in roots to discriminate between roots of different species growing adjacent to one another and hence be able to follow pH dynamics of roots whose species identity we knew.

Our setup had an intercropping factor with three levels: (i) one maize individual growing together with one bean seedling, (ii) one maize individual growing together with two bean seedlings, and (iii) a control level of one maize individual growing alone (in order to visualize rhizosphere pH dynamics without close contact between roots of the two species). The limited number of bean seedlings allowed no cultivation of single grown bean plants, therefore we used ROIs of bean roots growing without neighbors within the intercropping rhizotrons. So, for example, in **Figure 3**, the pH data depict values for maize from maize growing in its own rhizotron, whereas the pH data for bean depict values from a bean root growing without close neighbors but in an intercropped rhizotron. In intercropped rhizotrons we therefore had two ROIs with planar optodes attached, corresponding to maize root next to bean, bean root growing without a neighbor.

It is important to note that the spatial scale of a planar optode ROI is very much smaller than that of the whole rhizotron, such that we considered the ROIs as replicates in most cases [e.g., see **Figure 5**; see arguments in Hurlbert (1984) on the issue of spatial scale and pseudo-replication in experiments]. Seedlings were transplanted into four rhizotrons per factor level (i.e., *n* = 4) on Day 1.

Four days after transplanting (DAT), all roots had reached half way to the bottom of the rhizotrons. Planar optodes were placed into the rhizotrons on 5 DAT. The number of optodes was limited and therefore not all rhizotron replicates could be investigated at each time point: for the evaluation of pH dynamics particular ROIs within every single optode where determined according to the following scheme: central on surface of maize/bean root, bulk soil close to maize/bean root (i.e., 6–10 mm off the root surface) and bulk soil between the roots of both species.

Additionally, it turned out that after the placement of the optodes two intercropping rhizotrons could not be included in the further analysis because the roots of maize and bean grew together too close in order to separate individual pH signals.

During the course of the experiment, some ROIs showed an unexpectedly strong pH drop which was out of the range of the calibration curve (see chapter below). This caused a reduction of number of replicates during data analysis. In particular the number of replicates changed as follows: maize *n* = 4 DAT 6–8 and 14, *n* = 3 DAT 12; bean *n* = 4 DAT 6–8, *n* = 3 DAT 12–14; bulk soil close to bean/maize *n* = 4 DAT 6–14; bulk soil between roots *n* = 4 DAT 6–8, *n* = 2 DAT 12–14. Conventional and fluorescent pictures (for FP) as well as pH measurements using the planar optodes were taken on the following days of the experiment: 6 (morning and afternoon), 7 (afternoon), 8 (afternoon), 12 (afternoon), and 14 (morning).

### GFP TECHNIQUE

To identify the plant species crossing the optode region in a first step, the plant roots of maize and bean grown along the transparent plate of the rhizotrons were imaged with a conventional camera system and with an adapted lighting system-filtered camera to excite the FPs as described in Faget et al. (2009). In this paper, to adapt the previously developed method for minirhizotron to rhizotron with the transparent plate, we used a digital camera Canon, G10 mounted on a tripod. The conventional camera systems use ambient light and photograph the roots at the interface of the soil with the transparent plexiglass window of the rhizotrons. For the adaptation of this system to GFP, we mounted a filter (LONG 515 nm, Edmund Optics, Barrington, USA) in front of the camera to allow only roots expressing the GFP to be visible under excitation light (at wavelengths of 440–460 nm); further details including the components and standardized protocol are given in Faget et al. (2010).

At harvest, a closer identification was necessary to assess the root identity under the optode by removing the sensor and re-screening this area with conventional and fluorescent imaging techniques.

### OPTODE TECHNIQUE

Depending on the optical setup, different spatial and temporal resolutions can be achieved. In our experimental design we used the setup as recently described in Blossfeld et al. (2013). In particular, we used a fluorescent detection system with a field of view of 15 mm × 12 mm and a pixel resolution of 12 μm. In detail, this detection system is based on a modified USB-microscope device that consists of a light-emitting diode (LED) ring (470 nm) functioning as the excitation light source, filters, lens, and the complementary metal-oxide-semiconductor (CMOS) chip. The detection system is connected via USB to a PC and powered by this connection. Thus, this system is highly flexible and even portable, when using a notebook. The RGB images [24-bit, 1280 × 1024 (1.3 megapixel)] created by this detection system contain the raw, i.e., untreated sensor response. Hence, these red, green, blue (RGB) images needed to be analyzed with an image processing software (VisiSens; PreSens GmbH, Regensburg, Germany). This software calculates the ratio of red to green in the emitted fluorescence response (so-called *R*-value) provided by the color channels of the CMOS chip. This is possible because the optodes were made of two different dyes that are either analyte-sensitive or analyte-insensitive. The intensity of the green fluorescence of the analyte-sensitive dye is driven by the analyte concentration, whereas the intensity of the red fluorescence of the analyte-insensitive dye is not. The CMOS chip captured the red and green fluorescence in one single image and therefore the created *R*-value then provided a two-dimensional quantitative map of the measured parameter, i.e., the pH.

Several optode sensor foils (size 10 mm × 20 mm, product code SF-HP5-OIW; PreSens GmbH) were fixed at the transparent front plate of the rhizotrons with plants growing in them. The positioning was done 5 DAT when the roots had reached almost the lower third of the rhizotrons. By adding the planar optodes at this time point, we ensured that the placement of the planar optode was at a ROI (size 2 cm × 1 cm). We chose our ROIs in the following manner: we placed the optode on a zone where the tip of a growing root was just inside the area covered by the optode; this allowed for measurement of pH changes in most of the optode region without direct root contact at time point zero, as well as the dynamic measurement of pH changes as the root(s) grew through the ROI, i.e., behind the optode.

The rhizotrons were closed again after the placement of the planar optodes and first daily measurements were performed after one day of equilibration. The soil moisture and temperature was monitored in four rhizotrons via frequency domain reflectometry (FDR)-probes (Model: 5TE, Decagon Devices Inc., 2365 NE Hokins Court, Pullman WA 99163) parallel to the daily measurements and ranged between 26.6 and 36.3% (volumetric water content, VWC) as well as 24.3 and 25.1°C in the afternoon.

### CALIBRATION OF PLANAR OPTODES

Prior to the start of the experiment, the optical setup together with the planar optodes was calibrated. This was achieved by using a small transparent vessel containing defined pH buffer solutions (mixture of K_2_HPO_4_ and KH_2_PO_4_, controlled with standard pH glass electrodes) and a small replicate of the planar optode batch installed on the inside of this vessel.

**FIGURE 1:**
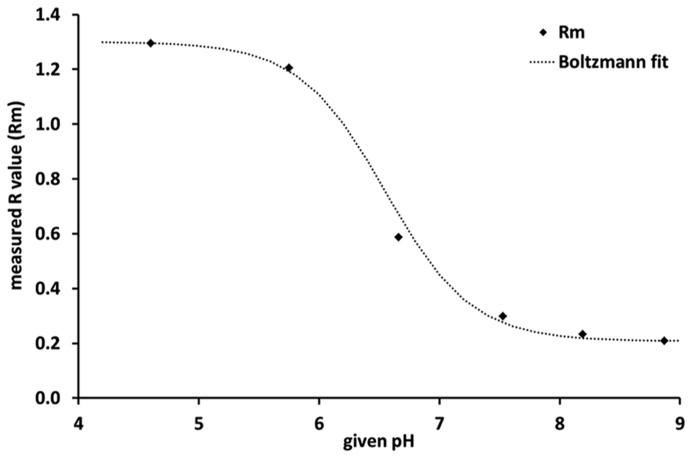
**Calibration curve of the planar optodes, where *R*_m_ is the measured *R*-value, i. e., the ratio of red to green in the emitted fluorescence response.** The steep slope of the Boltzmann fit between pH 6 and pH 7 indicates that the sensor is most sensitive within this pH range.

The average *R*-value (*R*_m_) of this replicate for each given pH was recorded and used as input parameter for a fitting function. The relationship between *R*_m_ and the given pH can be described by a sigmoidal Boltzmann equation (Eq. 1). This equation was adapted from (Blossfeld and Gansert, 2007) by exchanging the parameter π with the parameter *R*. This equation can be transformed in order to calculate the pH from the measured *R*-value during the experiment (Eq. 2). This equation was also adapted from Blossfeld and Gansert (2007) by exchanging the parameter π with the parameter *R*.

1 $$R_{m}=\frac{R_{\min}-R_{\max}}{\left\{1+Exp\left[(pH_{m}-pH_{0})dpH\right]\right\}}+R_{\max},\;$$

2 $${pH}_{m}={pH}_{0}+dpH\times In[\frac{\left(R_{\min}-R_{\max}\right)}{(R_{m}-R_{\max})-1}],$$

where *R*_m_ is the calculated/measured *R*-value, *R*_min_ and *R*_max_ represent the upper and lower range of the fitting; pH_0_ is the inflection point and dpH the slope of the fitted curve. The Boltzmann fit clearly demonstrates that the sensitivity of the sensor was highest between pH 6 and pH 7 and lowest below pH 5 and above pH 8 (**Figure 1**).

## RESULTS AND DISCUSSION

**FIGURE 2:**
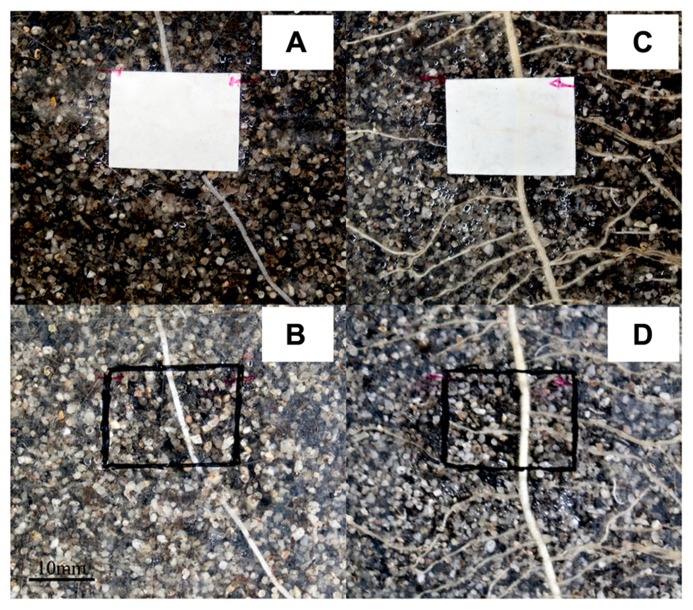
**Photographs of the experimental setup as seen through the transparent window of the rhizotrons with and without optodes installed.** The pictures show roots growing with no neighbor of another species nearby. Panels **(A,B)** are photographs of maize roots while **(C,D)** are of bean roots. Panels **(A,C)** were shot at the time of destructive harvesting which corresponded to DAT 14 (DAT, days after transplanting) and we can clearly see the roots crossing the planar optodes. The optodes where removed as seen in **(B,D)** to precisely locate the root trajectories under the sensors. The scale is given by the optodes which measure 10 mm × 20 mm regions of interest (ROIs).

**Figure 2** shows photographs through the transparent window of the rhizotrons of maize roots on the left side (**Figures 2A, B**) and bean roots on the right side (**Figures 2C, D**) growing alone with no neighbors in the proximal rhizosphere. The upper row (**Figures 2A, C**) was taken before harvest showing the position of the planar optodes on the root systems through the window interface. Just before harvest, the planar optodes were taken away to identify and measure the exact location of the roots behind the optode sensors (**Figures 2B, D**).

**FIGURE 3:**
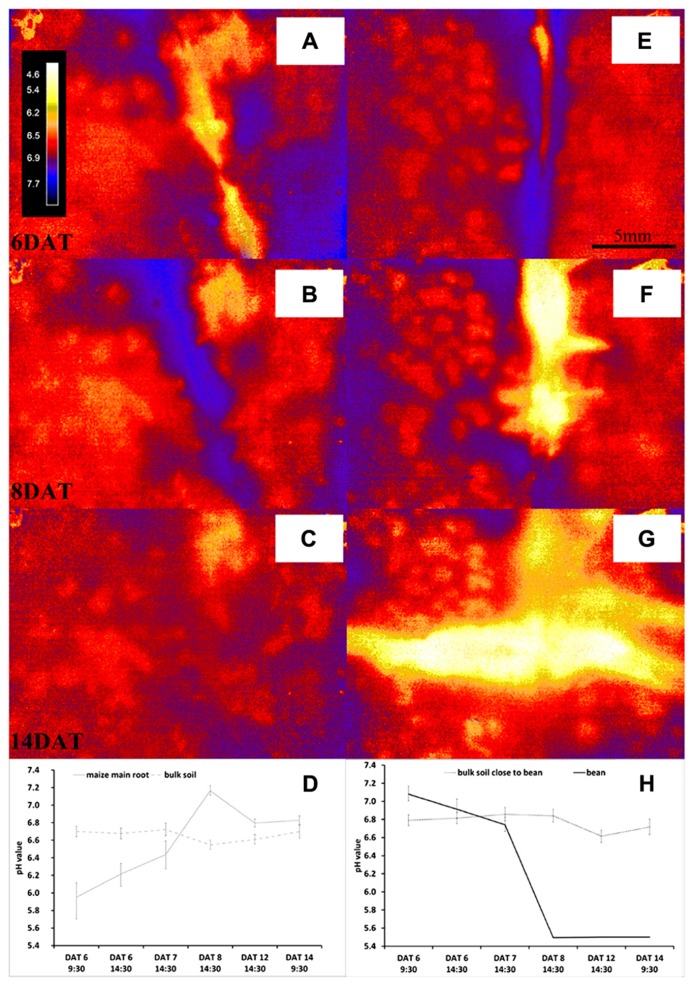
**Dynamics over time of pH measured with the optodes for the rhizosphere and bulk soil of roots of maize (A–D) or bean (E–H) growing alone.** Panels **(A–C,E–G)** show the pH maps of the respective ROIs at a scale ranging from 4.6 to 7.7 pH units at DAT 6 **(A,E)**, DAT 8 **(B,F)**, and DAT 14 **(C,G)**, respectively; the scale here is 20 mm × 10 mm. Panels **(D,H)** show the evolution of the mean pH value (±SD) of all pixel within the ROI at the root surface of maize **(D)** and bean **(H)** over time growing in separate rhizotrons.

The pH monitoring via the optodes revealed that the investigated species modified their rhizosphere pH creating very distinctive patterns; **Figure 3** shows the evolution of pH measured by the planar optodes over time. We found clear dynamic changes of pH over time and differences between the two species in rhizosphere acidification both when roots grew alone and in interaction between the species.

Initially, the roots of maize growing alone acidified the rhizosphere on average by 0.75 pH units compared to the bulk soil pH (**Figure 3A**). This rhizosphere acidification was not constant over time, but changed instead to a net alkalization of up to 0.62 pH units on 8 DAT (**Figure 3B**). In the later phase of the experiment the rhizosphere pH came closer to the bulk soil pH, which varied between pH 6.55 and pH 6.72 (**Figure 3C**).

Interestingly, the single grown bean roots showed the opposite behavior (**Figures 3D, H**). The rhizosphere of this young bean roots was 0.29 pH units higher than the initial bulk soil pH of 6.79 (**Figure 3E**). However, from 8 DAT onward, the bean roots acidified the rhizosphere in such a strong manner that the sensor signal was below pH 5.5 (**Figure 3F**). The young lateral roots of bean acidified the rhizosphere right from their emergence onward and it cannot be excluded that some of the acidic molecules diffused along the lateral roots to the main roots (**Figure 3G**). It should also be noted that the bean roots formed no nodules during the course of the experiment. Since both species were grown in the same substrate and all rhizotrons received the same watering regime with the same nutrient solution, this contrasting pattern is very interesting. Since the only source of nitrogen in the rhizosphere of all plants was derived from the nitrate of the nutrient solution, we expected that an uptake of this nitrate would cause an alkalization of the rhizosphere (Marschner and Romheld, 1983; Colmer and Bloom, 1998; Cousins and Bloom, 2003). This was what we found around the maize roots, but not the bean roots, although we cannot confirm with our study that this is the mechanism behind the pH patterns we found. The bean rhizosphere pH response is difficult to interpret given that there were no nodules on the roots and hence no sign of N_2_-fixation occurring, which would have potentially explained the acidification over time as protons are released during fixation (Bolan et al., 1991). Another explanation could be the species-specific ability to mobilize phosphorous (P) in the rhizosphere. It has been reported that under P-limitation but high nitrate content non-nodulated roots of faba bean heavily acidified the rhizosphere, whereas maize roots alkalinized their rhizosphere when growing under the same conditions (Li et al., 2007). However, we have not measured the P-content of the plants and the soil after the experiment in order to verify this explanation. Thus, the patterns found now need further testing with more replication, further soil, and plant analysis and with a variety of species in addition to maize and bean.

**FIGURE 4:**
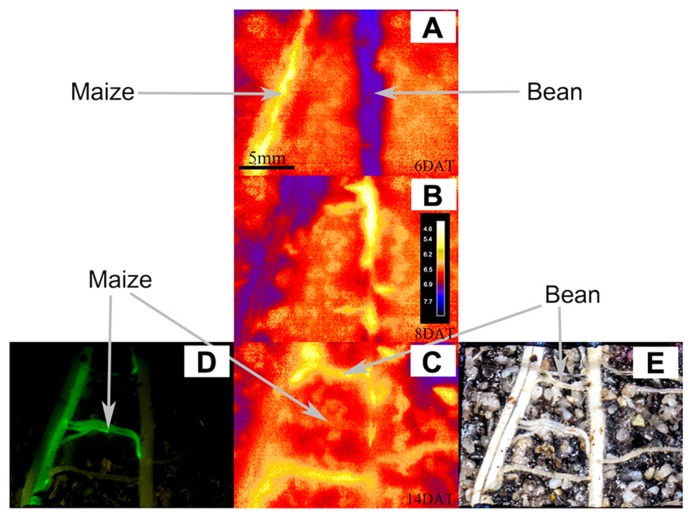
**This figure shows the potential of combining fluorescence (GFP) with optode pH methods, illustrating what each method can contribute to understanding who is who during rhizosphere pH changes.** The figure shows the ROIs for the rhizosphere and the bulk soil of roots of maize and bean growing in close proximity to each other. Panels **(A–C)** show the pH maps of pH measured with the optodes for the rhizosphere and bulk soil of roots of maize and bean growing in close proximity: at a scale ranging from 4.6 to 7.7 pH units at DAT 6 **(A)**, DAT 8 **(B)**, and DAT 14 at the end of the experiment **(C)**. Panels **(D,E)** show photographs of these same ROIs taken on DAT 14 at harvesting after having removed the optode for locating and identifying roots. Panel **(D)** was photographed under blue light to excite the maize expressing the GFP, allowing the identification and exact location of the maize roots (the roots tips and meristematic areas are even brighter than the remaining tissue). Panel **(E)** shows a conventional photograph that is complementary to **(D)** shot in conventional light, where all the roots form maize and bean are visible.

**FIGURE 5:**
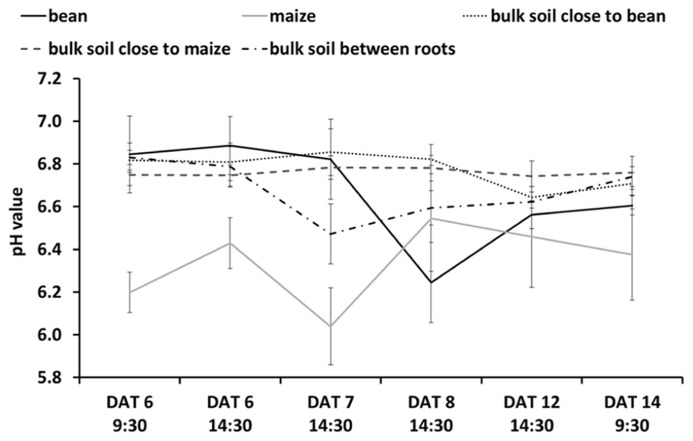
**pH Dynamics over time as related to different ROIs either in the rhizosphere of maize or bean (i. e., positioned centrally on the individual root), in the bulk soil close to maize or bean roots (i.e., positioned 6–10 mm away from the individual root), or in the bulk soil between maize and bean roots (i.e., positioned centrally between roots with >6 mm distance between them).** Values are means and standard errors of the mean of all pixels (approx. 2500–3500 pixels for the rhizosphere and 10000–15000 pixels for bulk soil) in the individual ROI. Note that these mean values are derived solely from intercropping rhizotrons, such that they are composed of values from rhizotrons with maize intercropped with one or with two bean individuals. For maize *n* = 4 DAT 6–8 and 14, *n* = 3 DAT 12; bean *n* = 4 DAT 6–8, *n* = 3 DAT 12–14; bulk soil close to bean/maize *n* = 4 DAT 6–14; bulk soil between roots *n* = 4 DAT 6–8, *n* = 2 DAT 12–14.

**Figure 4** shows the evolution of pH over time when roots of both species grew within the proximity of the other. **Figures 4A–C** show the variations in acidification and alkalization of the rhizosphere at 6, 8, and 14 DAT, respectively. **Figures 4D, E** allow us to identify which roots belonged to which species (using the GFP method and conventional photography): in interaction, we see an acidification, then alkalization followed by an acidification of the maize root, with a clear acidification of the bean root over time (**Figures 4A–C**). Overall (see **Figure 5** for more detailed views of the pH changes) we found that the pattern of rhizosphere acidification over time was similar to that found when the roots of one species were not in the proximity to the other, but the intensity of the pH changes was about 0.6 pH units lower.

We cannot yet explain why we found a less strong change in pH compared to bulk soil (**Figure 5**) when roots of the two species were directly interacting. Further studies should help identify whether this was due to the species interactions and some kind of plant–plant communication or other more resource-based competitive outcomes (see Faget et al., 2013 for discussion of this topic).

Without the GFP method it would have been impossible to distinguish by eye, which root belonged to which species and thus which pH activity could be assigned to the maize or to the bean root zones (as is the case in **Figures 2 and 3**). At harvest time (14 DAT) the optode was removed and the roots were imaged (**Figure 4E**). This conventional photograph is helpful to visualize the location of different roots behind the optodes but alone does not allow one to identify to which species they belong. By using the GFP method, as in this case maize roots expressing the GFP, it was possible to separate maize from bean roots and to then compare pH dynamics in specific ROIs.

**Figure 4D** clearly shows the maize roots in fluorescent green, differing from the bean roots in pale color or even not visible on the GFP-image but only on the conventional image. Here we can see that some of the lateral roots belonged to maize and some to bean, which would not have been visible to the naked eye. This then explains why not all visible lateral roots acidified their rhizosphere and why the acidification of the upper and lower lateral roots is not as prominent as in the single root observations (**Figure 5**).

In **Figure 4D** one can clearly see that only the acidifying roots belong to the bean plant and the central lateral roots belong to the maize plant. Hence, by combining the GFP method with the planar optode methods, it is now possible to follow the pH variation of the rhizosphere during plant–plant interactions and precisely indicate which species had what kind of influence on the rhizosphere properties, even if the mechanism behind the patterns requires further complementary studies.

Combining these methods should also allow one to compare the integrated effect of roots growing alone or with neighbors on rhizosphere pH (or O_2_ or CO_2_) with outcomes when roots are interacting directly. For example in our study, we found that the modification of the rhizosphere pH when roots of two species were directly interacting was similar to the roots growing alone (**Figure 5**). The maize still tended to alkalinize the rhizosphere and the bean still acidified it, but the intensity of the modification by the roots of both species was reduced.

We also found that the pH of the bulk soil in proximity of either the maize roots or bean roots did not show strong variation while the pH of the bulk soil in between the two roots systems suggests it may be an averaging of the pH values for roots growing alone.

Our approach has the potential to prove very useful in so-called *guided sampling*. High-throughput phenotyping of plant traits is currently a burgeoning field in plant sciences (Rascher et al., 2011; Nagel et al., 2012; Fiorani and Schurr, 2013), and allows for large screening of many genotypes and species. At times, high-throughput phenotyping can benefit greatly from more detailed lower-throughput methods such as the described planar optode method for studying processes in the rhizosphere at particular points in space or time deemed particularly interesting. The planar optode method can report differences in rhizosphere (metabolic) activity of different roots, including hotspots of root activity in the main or lateral roots at different times. Information derived from the optodes and the GFP-images could then be used directly for guided sampling of specific root/rhizosphere sections for analysis of compounds, enzymes, microbial communities, etc. One of the main limitations would come from the need to use genetic modified plant material. This is a pre-condition in order to be able to distinguish roots from different species. GFP-transformed *Arabidopsis thaliana* is readily available, whereas it is not available yet for many other plant species since transformation involves a considerable amount of work.

Another area of research where we deem that the application of these two methods may be very promising is in plant–plant interaction studies in ecology and ecophysiology. In these research fields, a range of different theories to explain patterns found in nature are being tested based on both resource-based and non-resource-based competition, novel communication pathways between plants (Zavala and Baldwin, 2006; Gagliano et al., 2012), as well as considering the role of positive interactions between plants as well as competition (Temperton et al., 2007; Brooker et al., 2008).

Not only GFP is available but different colors have now been made available in a number of mainly agriculturally interesting species which will make possible for us to be able to distinguish and study root–root interactions within populations as well as communities in the longer run. For example, maize expressing the GFP was combined with wheat expressing the RFP and rapeseed as wild type in Faget et al. (2013). At the same time, planar optodes can measure not only pH but also CO_2_, O_2_, and ammonium (Stromberg, 2008) and the size of the optodes available for research is increasing such that whole rhizotrons can soon follow plant–soil dynamics over time. This combination of novel methods for studying root biology and ecology should pave the way to an improved understanding of both root–soil and root–root interactions.

## Conflict of Interest Statement

The authors declare that the research was conducted in the absence of any commercial or financial relationships that could be construed as a potential conflict of interest.

## References

[B1] Blossfeld S. (2013). Light for the dark side of plant life: planar optodes visualizing rhizosphere processes. *Plant Soil* 369 29–32 10.1007/s11104-11013-11767-11100.

[B2] Blossfeld S. Gansert D. (2007). A novel non-invasive optical method for quantitative visualization of pH dynamics in the rhizosphere of plants. *Plant Cell Environ.* 30 176–186 10.1111/j.1365-3040.2006.01616.x 17238909

[B3] Blossfeld S. Gansert D. (2012). *The Use of Planar Optodes in Root Studies for Quantitative Imaging*. New York: Springer

[B4] Blossfeld S. Gansert D. Thiele B. Kuhn A. J. Losch R. (2011). The dynamics of oxygen concentration, pH value, and organic acids in the rhizosphere of *Juncus* spp. *Soil Biol. Biochem.* 43 1186–1197 10.1016/j.soilbio.2011.02.007

[B5] Blossfeld S. Schreiber C. M. Liebsch G. Kuhn A. J. Hinsinger P. (2013). Quantitative imaging of rhizosphere pH and CO_2_ dynamics with planar optodes. *Ann. Bot.* 112 267–276 10.1093/aob/mct047 23532048 PMC3698388

[B6] Bolan N. S. Hedley M. J. White R. E. (1991). Processes of soil acidification during nitrogen cycling with emphasis on legume based pastures. *Plant Soil* 134 53–63 10.1007/BF00010717

[B7] Bonkowski M. Villenave C. Griffiths B. (2009). Rhizosphere fauna: the functional and structural diversity of intimate interactions of soil fauna with plant roots. *Plant Soil* 321 213–233 10.1007/s11104-009-0013-2

[B8] Brooker R. W. Maestre F. T. Callaway R. M. Lortie C. L. Cavieres L. A. Kunstler G. (2008). Facilitation in plant communities: the past, the present, and the future. *J. Ecol.* 96 18–34 10.1111/j.1365-2745.2007.01295.x

[B9] Cahill J. F. Mcnickle G. G. Haag J. J. Lamb E. G. Nyanumba S. M Clair C. C. S. (2010). Plants integrate information about nutrients and neighbors. *Science* 328 1657–1657 10.1126/science.1189736 20576883

[B10] Callaway R. M. Brooker R. W. Choler P. Kikvidze Z. Lortie C. J. Michalet R. (2002). Positive interactions among alpine plants increase with stress. *Nature* 417 844–848 10.1038/nature00812 12075350

[B11] Colmer T. D. Bloom A. J. (1998). A comparison of ${NH}_{4}^{+}\;$ and ${NO}_{3}^{-}\;$ net fluxes along roots of rice and maize. *Plant Cell Environ.* 21 240–246 10.1046/j.1365-3040.1998.00261.x

[B12] Cousins A. B. Bloom A. J. (2003). Influence of elevated CO_2_ and nitrogen nutrition on photosynthesis and nitrate photo-assimilation in maize (*Zea mays* L.). *Plant Cell Environ.* 26 1525–1530 10.1046/j.1365-3040.2003.01075.x

[B13] Darwin C. R. (1880). *The Power** of Movements in Plants*. London: John Murray

[B14] de Kroon H. (2007). Ecology – how do roots interact? *Science* 318 1562–1563 10.1126/science.1150726 18063777

[B15] de Kroon H. Hendriks M. Van Ruijven J. Ravenek J. Padilla F. M. Jongejans E. (2012). Root responses to nutrients and soil biota: drivers of species coexistence and ecosystem productivity. *J. Ecol.* 100 6–15 10.1111/j.1365-2745.2011.01906.x

[B16] de Kroon H. Mommer L. (2006). Root foraging theory put to the test. *Trends Ecol. Evol.* 21 113–116 10.1016/j.tree.2005.11.021 16701484

[B17] Dudley S. A. File A. L. (2007). Kin recognition in an annual plant. *Biol. Lett.* 3 435–438 10.1098/rsbl.2007.0232 17567552 PMC2104794

[B18] Dudley S. A. Murphy G. P. File A. L. (2013). Kin recognition and competition in plants. *Funct. Ecol.* 27 898–906 10.1111/1365-2435.12121

[B19] Faget M. Herrera J. M. Stamp P. Aulinger-Leipner I. Frossard E. Liedgens M. (2009). The use of green fluorescent protein as a tool to identify roots in mixed plant stands. *Funct. Plant Biol.* 36 930–937 10.1071/FP09125 32688704

[B20] Faget M. Liedgens M. Feil B. Stamp P. Herrera J. M. (2012). Root growth of maize in an Italian ryegrass living mulch studied with a non-destructive method. *Eur. J. Agron.* 36 1–8 10.1016/j.eja.2011.08.002

[B21] Faget M. Liedgens M. Stamp P. Flutsch P. Herrera J. M. (2010). A minirhizotron imaging system to identify roots expressing the green fluorescent protein. *Comput. Electron. Agric.* 74 163–167 10.1016/j.compag.2010.06.010

[B22] Faget M. Nagel K. A. Walter A. Herrera J. M. Jahnke S. Schurr U. (2013). Root–root interactions: extending our perspective to be more inclusive of the range of theories in ecology and agriculture using *in vivo* analyses. *Ann. Bot.* 112 253–266 10.1093/aob/mcs296 23378521 PMC3698385

[B23] Fang C. X. Zhuang Y. E. Xu T. C. Li Y. Z. Li Y. Lin W. X. (2013). Changes in rice allelopathy and rhizosphere microflora by inhibiting rice phenylalanine ammonia-lyase gene expression. *J. Chem. Ecol.* 39 204–212 10.1007/s10886-013-0249-4 23385369

[B24] Fiorani F. Schurr U. (2013). Future scenarios for plant phenotyping. *Annu. Rev. Plant. Biol.* 64 267–291 10.1146/annurev-arplant-050312-120137 23451789

[B25] Gagliano M. Renton M. Duvdevani N. Timmins M. Mancuso S. (2012). Out of sight but not out of mind: alternative means of communication in plants. *PLoS ONE* 7:e37382 10.1371/journal.pone.0037382 PMC3358309 22629387

[B26] Gansert D. Blossfeld S. (2008). “The application of novel optical sensors (optodes) in experimental plant ecology,” in *Progress in Botany* eds Lüttge U. Beyschlag W. Murata J. (Berlin: Springer) 333–358 10.1007/978-3-540-72954-9_14

[B27] Hiltner L. (1904). über neuere Erfahrungen und Probleme auf dem Gebiet der Bodenbakteriologie und unter besonderer Berücksichtigung der Grunddüngung und Brache. *Arb. Deutsche. Landwirt. Ges.* 98 59–78

[B28] Hinsinger P. Bengough A. G. Vetterlein D. Young I. M. (2009). Rhizosphere: biophysics, biogeochemistry and ecological relevance. *Plant Soil* 321 117–152 10.1007/s11104-008-9885-9

[B29] Hinsinger P. Gobran G. R. Gregory P. J. Wenzel W. W. (2005). Rhizosphere geometry and heterogeneity arising from root-mediated physical and chemical processes. *New Phytol.* 168 293–303 10.1111/j.1469-8137.2005.01512.x 16219069

[B30] Hinsinger P. Plassard C. Tang C. X. Jaillard B. (2003). Origins of root-mediated pH changes in the rhizosphere and their responses to environmental constraints: a review. *Plant Soil* 248 43–59 10.1023/A:1022371130939

[B31] Hodge A. (2004). The plastic plant: root responses to heterogeneous supplies of nutrients. *New Phytol.* 162 9–24 10.1111/j.1469-8137.2004.01015.x

[B32] Holst G. Grunwald B. (2001). Luminescence lifetime imaging with transparent oxygen optodes. *Sens. Actuators B Chem.* 74 78–90 10.1016/S0925-4005(00)00715-2

[B33] Hurlbert S. H. (1984). Pseudoreplication and the design of ecological field experiments. *Ecol. Monogr.* 54 187–211 10.2307/1942661

[B34] Jones D. L. Dennis P. G. Owen A. G Van Hees P. A. W. (2003). Organic acid behavior in soils – misconceptions and knowledge gaps. *Plant Soil* 248 31–41 10.1023/A:1022304332313

[B35] Klemens J. A. (2008). Kin recognition in plants? *Biol. Lett.* 4 67–68 10.1098/rsbl.2007.0518 18089522 PMC2412940

[B36] Lambers H. Shane M. W. Cramer M. D. Pearse S. J. Veneklaas E. J. (2006). Root structure and functioning for efficient acquisition of phosphorus: matching morphological and physiological traits. *Ann. Bot.* 98 693–713 10.1093/aob/mcl114 16769731 PMC2806175

[B37] Li L. Li S.-M. Sun J.-H. Zhou L.-L. Bao X.-G. Zhang H.-G. (2007). Diversity enhances agricultural productivity via rhizosphere phosphorus facilitation on phosphorus-deficient soils. *Proc. Natl. Acad. Sci. U.S.A.* 104 11192–11196 10.1073/pnas.0704591104 17592130 PMC1899187

[B38] Marschner H. Romheld V. (1983). *In vivo* measurement of root-induced pH changes at the soil-root interface: effect of plant species and nitrogen source. *Z. Pflanzenphysiol.* 111 241–251

[B39] Nagel K. A. Putz A. Gilmer F. Heinz K. Fischbach A. Pfeifer J. (2012). GROWSCREEN-Rhizo is a novel phenotyping robot enabling simultaneous measurements of root and shoot growth for plants grown in soil-filled rhizotrons. *Funct. Plant Biol.* 39 891–904 10.1071/FP12023 32480839

[B40] Postma J. A. Lynch J. P. (2012). Complementarity in root architecture for nutrient uptake in ancient maize/bean and maize/bean/squash polycultures. *Ann. Bot.* 110 521–534 10.1093/aob/mcs082 22523423 PMC3394648

[B41] Rascher U. Blossfeld S. Fiorani F. Jahnke S. Jansen M. Kuhn A. J. (2011). Non-invasive approaches for phenotyping of enhanced performance traits in bean. *Funct. Plant Biol.* 38 968–983 10.1071/FP11164 32480955

[B42] Rewald B. Meinen C. Trockenbrodt M. Ephrath J. E. Rachmilevitch S. (2012). Root taxa identification in plant mixtures – current techniques and future challenges. *Plant Soil* 359 165–182 10.1007/s11104-012-1164-0

[B43] Schreiber C. M. Zeng B. Temperton V. M. Rascher U. Kazda M. Schurr U. (2011). Dynamics of organic acid occurrence under flooding stress in the rhizosphere of three plant species from the water fluctuation zone of the Three Gorges Reservoir, P.R. China. *Plant Soil* 344 111–129 10.1007/s11104-011-0732-z

[B44] Stahl H. Glud A. Schroder C. R. Klimant I. Tengberg A. Glud R. N. (2006). Time-resolved pH imaging in marine sediments with a luminescent planar optode. *Limnol. Oceanogr. Methods* 4 336–345 10.4319/lom.2006.4.336

[B45] Stromberg N. (2008). Determination of ammonium turnover and flow patterns close to roots using Imaging optodes. *Environ. Sci. Technol.* 42 1630–1637 10.1021/es071400q 18441813

[B46] Temperton V. M. Mwangi P. N. Scherer-Lorenzen M. Schmid B. Buchmann N. (2007). Positive interactions between nitrogen-fixing legumes and four different neighbouring species in a biodiversity experiment. *Oecologia* 151 190–205 10.1007/s00442-006-0576-z 17048010

[B47] Zavala J. A. Baldwin I. T. (2006). Jasmonic acid signalling and herbivore resistance traits constrain regrowth after herbivore attack in Nicotiana attenuata. *Plant Cell Environ.* 29 1751–1760 10.1111/j.1365-3040.2006.01551.x 16913864

